# Posterior Cerebral Infarction following Loss of Guide Wire

**DOI:** 10.1155/2013/164710

**Published:** 2013-12-08

**Authors:** Jean-Marc Bugnicourt, Denis Belhomme, Bruno Bonnaire, Jean-Marc Constans, Cécile Manaouil

**Affiliations:** ^1^Department of Neurology and Laboratoire de Neurosciences Fonctionnelles et Pathologies (UMR CNRS 8160), Amiens University Hospital, Place Victor Pauchet, 80054 Amiens Cedex 1, France; ^2^Department of Vascular Surgery, Amiens University Hospital, Place Victor Pauchet, 80054 Amiens Cedex 1, France; ^3^Department of Radiology, Amiens University Hospital, Place Victor Pauchet, 80054 Amiens Cedex 1, France; ^4^Forensic and Medical Law Unit, Amiens University Hospital, Place Victor Pauchet, 80054 Amiens Cedex 1, France

## Abstract

Stroke after internal jugular venous cannulation typically leads to acute carotid or vertebral arteries injury and cerebral ischemia. We report the first case of delayed posterior cerebral infarction following loss of guide wire after left internal jugular venous cannulation in a 46-year-old woman with a history of inflammatory bowel disease. Our observation highlights that loss of an intravascular guide wire can be a cause of ischemic stroke in patients undergoing central venous catheterization.

## 1. Introduction

Inadequate placement of a jugular venous catheter is a well-known complication, with serious and immediate secondary complications including stroke. Acute complications are usually associated with injury to contiguous structures [1], leading to carotid [2–6] or vertebral [7–9] arteries thrombosis and cerebral ischemia [2–4]. Here we present the first case of delayed posterior cerebral infarction following loss of guide wire after left internal jugular venous cannulation.

## 2. Case Presentation

**Figure 1:**
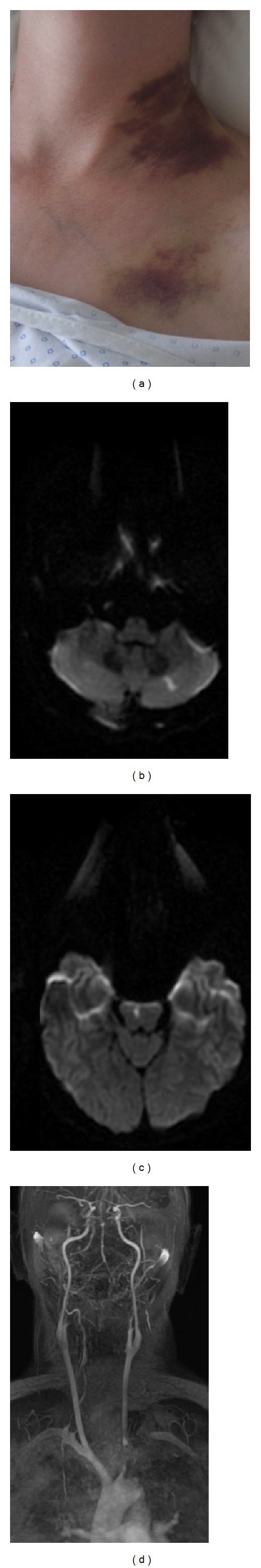
(a) Presence of a left supraclavicular subcutaneous hematoma. ((b), (c)) Diffusion-weighted magnetic resonance imaging showing left cerebellar (b) and right anterior pontine (c) hyperintensities related to limited infarcts in the posterior circulation territory. (d) Magnetic resonance angiography showing only the distal segment (V_4_) of the left vertebral artery.

**Figure 2:**
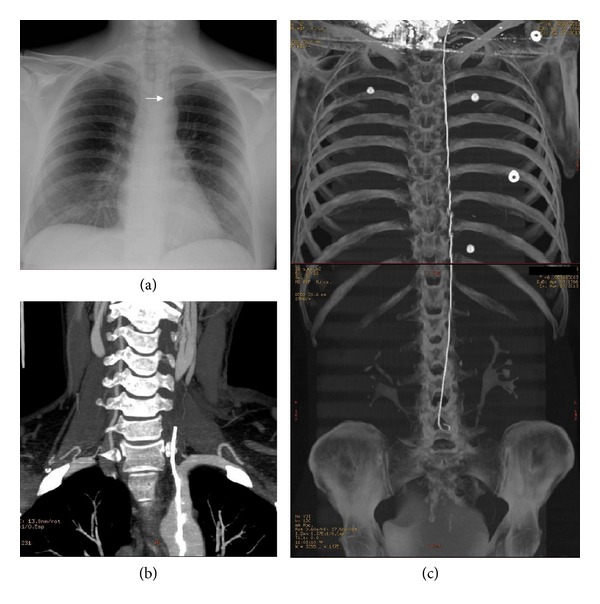
(a) Chest X-ray revealing the presence of the guide wire in the left hemithorax (arrow). (b) Cervical CT angiography showing insertion of the guide wire in the left subclavian artery, causing left vertebral artery occlusion, with the tip inserted in the superficial neck tissues. (c) Total body CT showing the vascular course of the guide wire as far as the distal abdominal aorta.

A 46-year-old woman with a history of inflammatory bowel disease was admitted for recurrence of severe active colitis and treated by intravenous corticosteroids. Left internal jugular venous cannulation was performed for total parenteral nutrition using the Seldinger technique. During catheterization, the patient suddenly experienced transient dizziness and blurred vision. No further problems were observed after withdrawing the catheter. The anaesthetist did not report any inattention during the procedure. Five days after hospital discharge, the patient presented sudden onset of difficult swallowing. Neurological examination revealed left hemiataxia with hypermetria, left Horner's sign, right deviation of the uvula indicating left palatal palsy, and hypoesthesia of the left hemiface. General examination revealed left cervical subcutaneous hematoma with no hemodynamic impairment (Figure 1(a)). Brain magnetic resonance imaging showed multiple acute cerebral infarcts in the vertebrobasilar territory (Figures 1(b) and 1(c)) and magnetic resonance angiography showed left vertebral artery occlusion from its origin (segment V_0_) to segment V_3_ with no signs of arterial dissection (Figure 1(d)). A lost guide wire was suspected on chest radiography (Figure 2(a)). Cervical CT angiography showed that the guide wire had been inadvertently inserted into the left subclavian artery, causing left vertebral artery occlusion. The upper extremity of the guide wire was located in the extravascular cervical region (Figure 2(b)), and total body CT scan showed the intravascular course of the guide wire as far as the abdominal aorta (Figure 2(c)). The guide wire was removed by exploration of the right femoral artery under general anaesthesia. The patient was discharged 7 days after admission with a favorable outcome.

## 3. Discussion

Cases of acute stroke caused by vertebral intimal damage with thrombosis leading to posterior cerebral infarction during internal jugular venous cannulation have been previously reported [7–9], although injury to the carotid artery is usually more frequent [2–6]. To the best of our knowledge, this is the first case to document delayed vertebral artery thrombosis resulting from loss of guide wire after central venous cannulation. Loss of a complete guide wire usually does not cause symptoms [10]. In our observation, potential causes of this ischemic stroke were left vertebral artery occlusion and foreign body embolism in a patient who was otherwise predisposed to hypercoagulable state. Loss of an intravascular guide wire is a very rare but completely preventable complication after Seldinger technique. Reported predisposing factors include inattention, operator inexperience, and overtired and rushed medical staff [10, 11].

In conclusion, our observation highlights that stroke after catheter placement is scarce but constitutes a major and potentially fatal complication of a benign condition.
